# Visual analysis of research hotspots and trends of external therapies in traditional Chinese medicine for chronic pain

**DOI:** 10.3389/fmed.2025.1712013

**Published:** 2026-01-14

**Authors:** Lingzu Kong, Xichen Wang, Jinying Zhao, Lijia Chai, Yinghua Hu, Fuchun Wang

**Affiliations:** 1 College of Acupuncture and Tuina, Changchun University of Chinese Medicine, Changchun, Jilin, China; 2 School of Medical Information, Changchun University of Chinese Medicine, Changchun, Jilin, China

**Keywords:** chronic pain, CiteSpace, external therapies in traditional Chinese medicine, research hotspots, research trends, visual analysis

## Abstract

**Objective:**

We aimed to explore the current research status and development trend of chronic pain treated with external therapies in traditional Chinese medicine (TCM) and to provide a reference for further research in this field.

**Methods:**

From the China National Knowledge Infrastructure, Web of Science, and PubMed databases, relevant articles on external therapies in TCM for chronic pain were selected as the research objects, and CiteSpace performed the bibliometric analysis.

**Results:**

A total of 3,220 English and 628 Chinese articles were included after CiteSpace was used to remove duplicate articles and perform manual screening. The English articles were analyzed; the core author is Witt, Claudia M, and the institution with the highest article production is Harvard University. The Chinese articles were analyzed, the core author is Fang Jianqiao, and the institution with the highest article production is Shanghai University of Traditional Chinese Medicine. The overall volume of publications is on an upward trend. Keyword analysis concluded that the research hotspots in this field were electroacupuncture therapy and tuina therapy, as well as common chronic pain-related diseases, and the research trends were cupping therapy, warm acupuncture therapy, and wrist and ankle acupuncture therapy, as well as chronic pain accompanied by depression.

**Conclusion:**

The results of this study suggested the relevant literature on external therapies in TCM for chronic pain. It highlights the direction for further exploration by revealing and analyzing the research hotspots and trends in this field.

## 1 Introduction

The International Association for the Study of Pain (IASP) defines chronic pain as pain that persists or recurs for more than 3 months. In recent years, the number of patients with this disease has increased. According to epidemiological surveys, the prevalence of chronic pain is 20.4%. At the same time, this disease increases the financial and psychological burden on patients, leading to a decline in their overall quality of life (1, 2). Studies have shown that there is a link between the intensity of chronic pain and sleep rhythms (3). Psychological surveys have shown that chronic pain patients score higher on depression and anxiety scales (4). In treatment regimens, opioid peptides are widely used in clinical practice due to their sound analgesic effects, with their usage rate increasing from 19.3 to 29.1% (5). However, given its side effects, such as dependence and addiction, strict control of the single dose and frequency of use of the drug is necessary (6, 7).

Traditional Chinese medicine external therapy generally refers to methods of treatment guided by traditional Chinese medical theory, involving the application of herbal medicines, techniques, or instruments to the skin (mucous membranes) of the body surface. This includes various methods such as acupuncture, moxibustion, friction, massage, electroacupuncture, cupping, and application of herbal plasters to acupoints. The advantages of traditional Chinese medicine external therapy in treating chronic pain conditions are primarily reflected in the following three aspects: first, its analgesic effects (8, 9); second, its effect on secondary depression and anxiety (10); third, minimal side effects and readily acceptable by patients.

Bibliometrics can be used to quantitatively analyze existing literature and identify key information in relevant fields. There is a large amount of complex literature on the use of traditional Chinese external therapies for the treatment of chronic pain. No researchers have conducted a comprehensive bibliometric analysis of this field. This paper employed CiteSpace, a scientific software, to analyzing the relevant literatures on using traditional Chinese external therapies to treat chronic pain (11, 12). CiteSpace is a visualization software developed by Prof. Chaomei Chen’s team based on the Java platform, which explores the evolution of a scientific domain, ranging from a single specialty to multiple interrelated scientific frontiers (13). Visualization analysis is an analytical approach that presents research data comprehensively and intuitively in the form of images. It is widely applied across various fields (14, 15). This study aims to enable researchers to grasp the basic knowledge system quickly and the current research status of traditional Chinese external therapies for chronic pain through visualization, while also guiding future research.

## 2 Materials and methods

### 2.1 Documentation sources

Computerized search databases included China National Knowledge Infrastructure (CNKI), PubMed, and Web of Science (WOS) in this review. Data on external TCM therapies for depression published between January 2005 and January 2025 were collected. The detailed search terms and strategies are presented in Table 1.

**Table 1:** Search strategies.

Database	Search term
PubMed	(((((((((((((((((Chronic pain[Title/Abstract]) OR (Chronic primary pain[Title/Abstract])) OR (primary chronic pain[Title/Abstract])) OR (Chronic primary visceral pain[Title/Abstract])) OR (Chronic widespread pain[Title/Abstract])) OR (Chronic primary musculoskeletal pain[Title/Abstract])) OR (Chronic primary headache[Title/Abstract])) OR (Chronic primary facial pain[Title/Abstract])) OR (Complex regional pain syndrome[Title/Abstract])) OR (chronic secondary pain[Title/Abstract])) OR (Chronic cancer related pain[Title/Abstract])) OR (Chronic postsurgical[Title/Abstract] OR post traumatic pain[Title/Abstract])) OR (Chronic secondary musculoskeletal pain[Title/Abstract])) OR (Chronic secondary visceral pain[Title/Abstract])) OR (Chronic neuropathic pain[Title/Abstract])) OR (Chronic secondary headache[Title/Abstract])) OR (Chronic secondary facial pain[Title/Abstract]) AND (((((((((((((((((((acupuncture[Title/Abstract]) OR (electric acupuncture[Title/Abstract])) OR (silver needle[Title/Abstract])) OR (tuina[Title/Abstract])) OR (moxibustion[Title/Abstract])) OR (moxa[Title/Abstract])) OR (cupping therapy[Title/Abstract])) OR (cupping[Title/Abstract])) OR (gua sha[Title/Abstract])) OR (rub sha[Title/Abstract])) OR (scrapping therapy[Title/Abstract])) OR (auricular-plaster[Title/Abstract])) OR (auricular point sticking[Title/Abstract])) OR (auricular point pressing[Title/Abstract])) OR (ear acupoint[Title/Abstract])) OR (acupoint application[Title/Abstract])) OR (acupressure points[Title/Abstract])) OR (acupuncture point applying[Title/Abstract])) OR (external therapies in traditional Chinese medicine[Title/Abstract])
Web of science	#1: TS = (Chronic pain) #2: TS = (Chronic primary pain) #3: TS = (primary chronic pain) #4: TS = (Chronic primary visceral pain) #5: TS = (Chronic widespread pain) #6: TS = (Chronic primary musculoskeletal pain) #7: TS = (Chronic primary headache) #8: TS = (Chronic primary facial pain) #9: TS = (Complex regional pain syndrome) #10: TS = (chronic secondary pain) #11: TS = (Chronic cancer related pain) #12: TS = (Chronic postsurgical or post traumatic pain) #13: TS = (Chronic secondary musculoskeletal pain) #14: TS = (Chronic secondary visceral pain) #15: TS = (Chronic neuropathic pain) #16: TS = (Chronic secondary headache) #17: TS = (Chronic secondary facial pain) #18: #17 OR #16 OR #15 OR #14 OR #13 OR #12 OR #11 OR #10 OR #9 OR #8 OR #7 OR #6 OR #5 OR #4 OR #3 OR #2 OR #1 #19: TS = (acupuncture) #20: TS = (electric acupuncture) #21: TS = (silver needle) #22: TS = (tuina) #23: TS = (moxibustion) #24: TS = (moxa) #25: TS = (cupping therapy) #26: TS = (cupping) #27: TS = (gua sha) #28: TS = (rub sha) #29: TS = (scrapping therapy) #30: TS = (auricular-plaster) #31: TS = (auricular point sticking) #32: TS = (auricular point pressing) #33: TS = (ear acupoint) #34: TS = (acupoint application) #35: TS = (acupressure points) #36: TS = (acupuncture point applying) #37: TS = (external therapies in traditional Chinese medicine) #38: #19 OR #20 OR #21 OR #22 OR #23 OR #24 OR #25 OR #26 OR #27 OR #28 OR #29 OR #30 OR #31 OR #32 OR #33 OR #34 OR #35 OR #36 OR #37 #39: #38 AND #18
CNKI	#1: SU % = ‘慢性疼痛’ OR SU % = ‘慢性原发性疼痛’ OR SU % = ‘慢性原发性内脏痛’ OR SU % = ‘慢性弥漫性疼痛’ OR SU % = ‘慢性原发性肌肉骨骼疼痛’ OR SU % = ‘慢性原发性头痛’ OR SU % = ‘慢性原发性口面部疼痛’ OR SU % = ‘复杂性区域疼痛综合征’ OR SU % = ‘慢性继发性疼痛’ OR SU % = ‘慢性癌症相关性疼痛’ OR SU % = ‘慢性术后或创伤后疼痛’ OR SU % = ‘慢性继发性肌肉骨骼疼痛’ OR SU % = ‘慢性继发性内脏痛’ OR SU % = ‘慢性神经病理性疼痛’ OR SU % = ‘慢性继发性头痛’ OR SU % = ‘慢性继发性口面部疼痛’ #2: SU % = ‘针刺’ OR SU % = ‘针灸’ OR SU % = ‘推拿’ OR SU % = ‘手法’ OR SU % = ‘按摩’ OR SU % = ‘埋线’ OR SU % = ‘火罐’ OR SU % = ‘刮痧’ OR SU % = ‘耳穴压豆’ OR SU % = ‘穴位贴敷’ OR SU % = ‘中医外治法’ #3#1 AND #2

### 2.2 Article screening criteria

#### 2.2.1 Inclusion criteria

The inclusion criteria were as follows: (i) articles relating to TCM external therapies for chronic pain; (ii) journal articles; and (iii) articles with complete information, including publication date, authors, and keywords.

#### 2.2.2 Exclusion criteria

The exclusion criteria were as follows: (i) repeatedly published literature, (ii) literature with research subject headings unrelated to external therapies in TCM for chronic pain, and (iii) conference papers, patents, news, advertisements, and popular science articles.

### 2.3 Data analysis

The data of the CNKI database were exported in “RefWorks” format, and the database in “RefWorks” format was imported into the built-in file format converter of the CiteSpace software. The database in “RefWorks” format was imported into the built-in file format converter of the CiteSpace software and converted into download_. txt format for use. The data from the WOS and PubMed databases were exported in the plain text format of WOS, and the checking and organizing function of the CiteSpace software was used to check and organize the articles of the two databases. The data were imported into CiteSpace, and the following parameters were set: time partition, 2004–2024; time slice (year per slice), “1”; and node types, author, institution, and keyword. In this way, a knowledge graph analysis of the articles’ author, institution, country, keyword, and other aspects was realized (13, 16, 17).

## 3 Results

### 3.1 Visual mapping of annual publication volume

**Figure 1:**
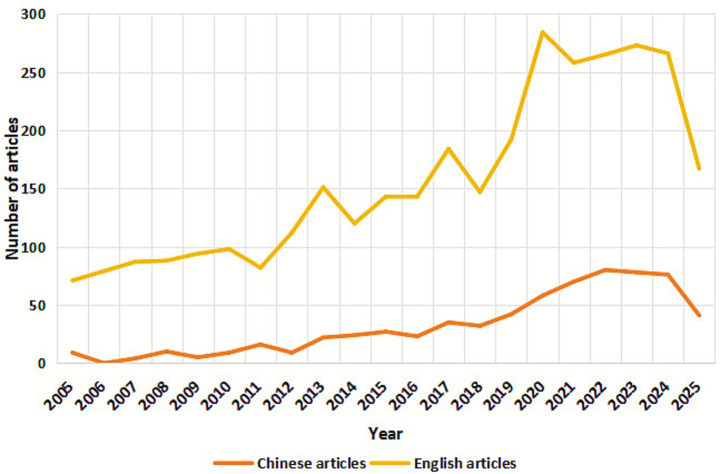
Annual articles of TCM external therapies for chronic pain.

The number of annual publications is an essential indicator for summarizing past research and predicting future research trends in this field. CiteSpace was used to eliminate duplicates and analyze the final 628 Chinese and 3,220 English articles (Figure 1).

### 3.2 Visual mapping of author publication volume

Using CiteSpace to analyze the authors associated with the Chinese and English literature on external therapies in TCM for chronic pain. We can identify the core research authors in this field and understand cooperation among authors. The size of the nodes in the network map represents the number of articles published by the authors, and the thickness of the lines reflects the intensity of collaboration between authors. Simultaneously, the analysis allows us to identify information about influential research groups and potential co-authors, thus helping researchers establish a network of collaborative relationships.

**Figure 2:**
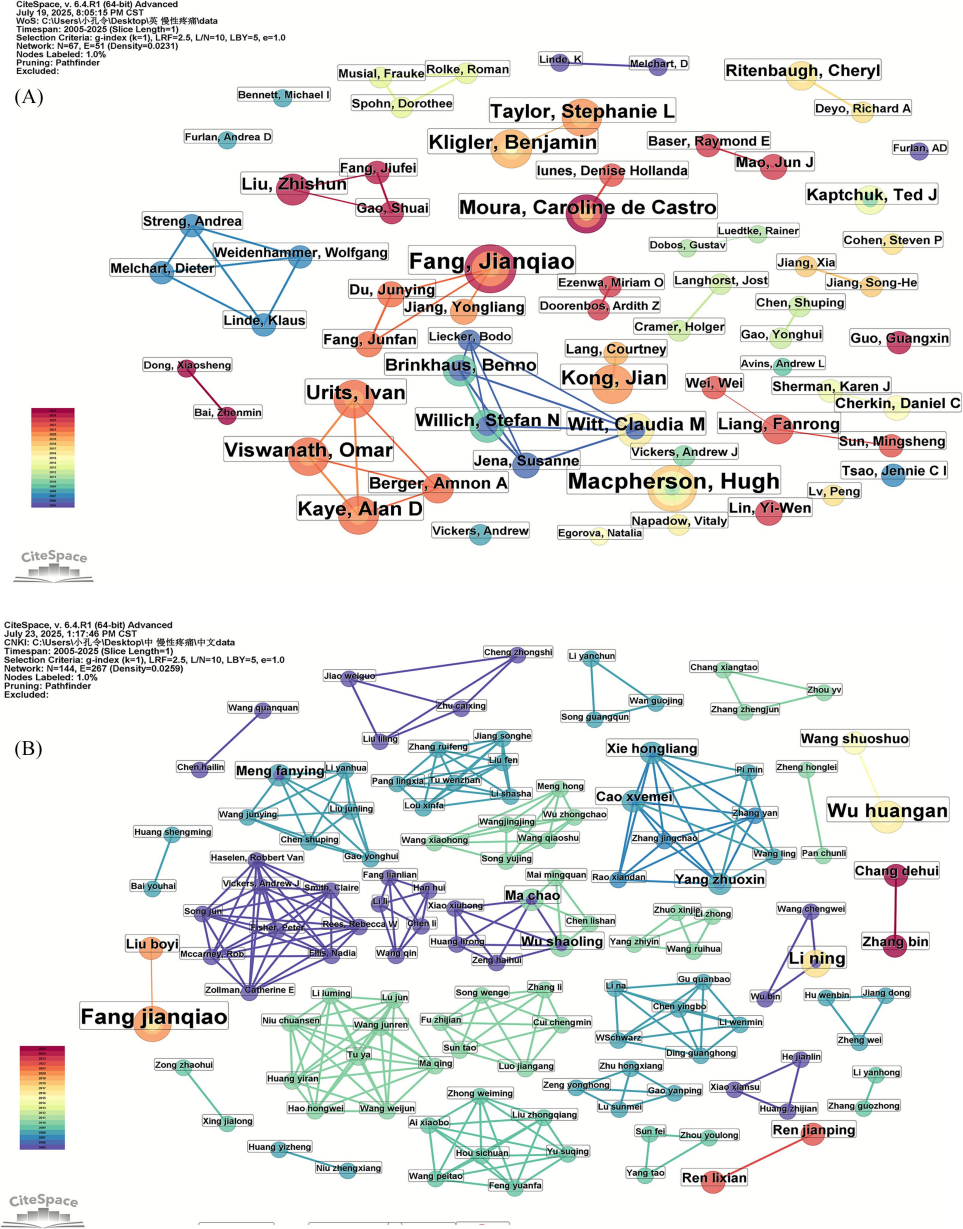
**(A)** Network map of authors of English literature; **(B)** network map of authors of Chinese literature.

The authors of the Chinese and English literature were analyzed using the CiteSpace software to generate an author collaboration network (Figures 2A,B) and to list the top 10 authors in terms of the number of articles published (Table 2). In the network map of the authors of the English literature (Figure 2A), the network temporal node was 67, the network temporal connectivity was 51, and the network density was 0.0231. In the network map of the authors of the Chinese literature (Figure 2B), the network time node was 144, the network time connectivity was 267, and the network density was 0.0259.

**Table 2:** The top 10 authors publishing articles.

No.	English literature		Chinese literature	
	Author	Frequency	Author	Frequency
1	Witt, Claudia M	47	Fang Jianqiao	10
2	Macpherson, Hugh	43	Kong Lingjun	6
3	Kong, Jian	38	Fang Min	6
4	Fang, Jianqiao	33	Liu Junling	6
5	Kligler, Benjamin	32	Wang Xin	6
6	Sherman, Karen J	31	Wang Xiyou	5
7	Napadow, Vitaly	30	Zhu Qingguang	5
8	Linde, Klaus	30	Liang Yi	5
9	Lin, Yi-Wen	28	Cheng Yanbin	5
10	Kaptchuk, Ted J	26	Shao Xiaomei	5

### 3.3 Visual mapping of institutional publication volume

Using CiteSpace for institutional analysis, we can identify the core research institutions in this field and understand cooperation among institutions. The size of the nodes in the network map of institutions represents the number of articles issued by the institutions, and the connecting lines reflect the strength of the inter-institutional cooperation relationship. This map facilitates researchers in quickly identifying potential collaborative institutions. The sharing of resources and knowledge among institutions contributes to advancing the field of study.

**Figure 3:**
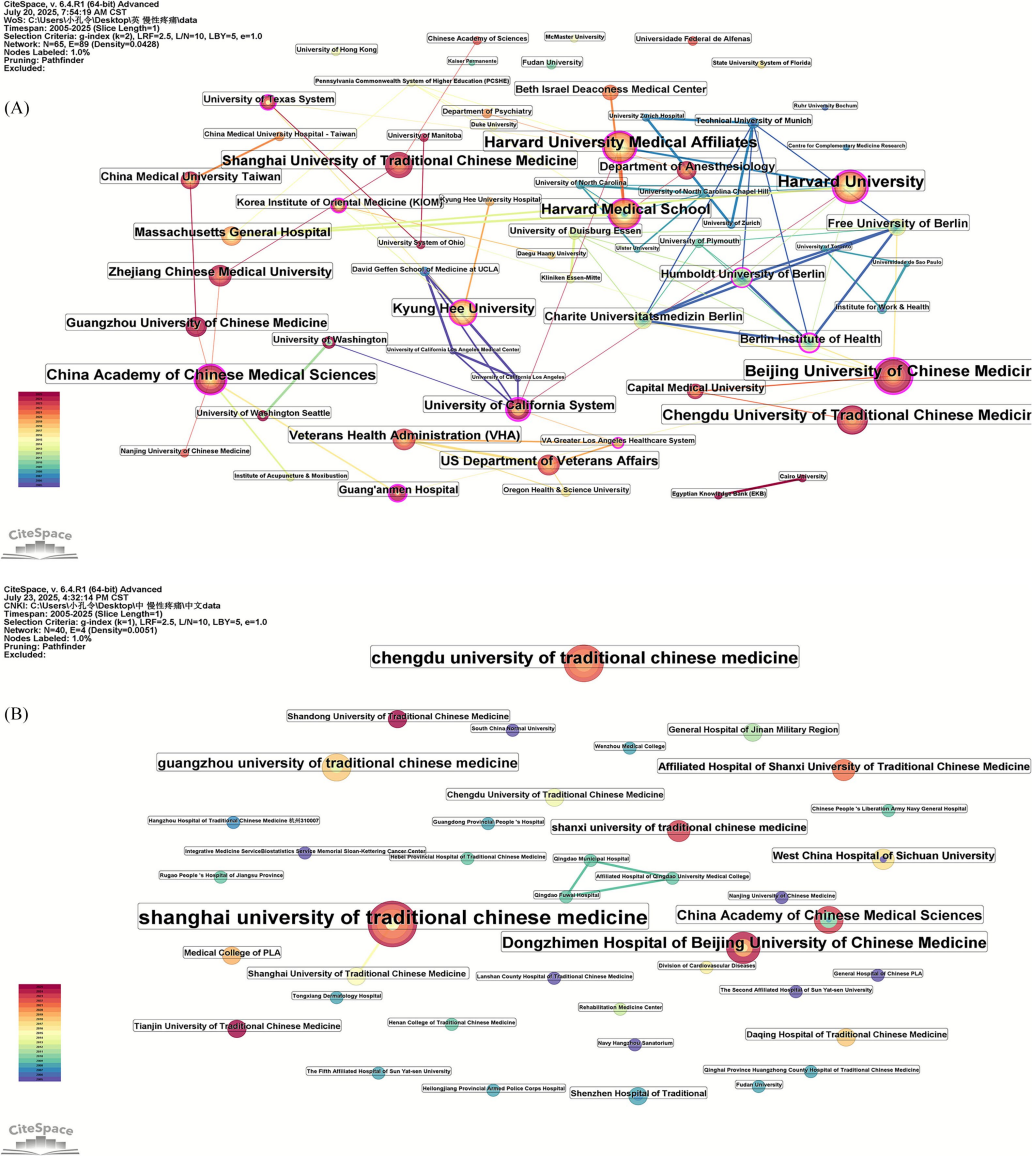
**(A)** Network map of institutions of English literature; **(B)** network map of institutions of Chinese literature.

The institutions of Chinese and English literature were analyzed using the CiteSpace software to generate an institution collaboration network (Figures 3A,B) and to list the top 10 institutions in terms of the number of articles published (Table 3). In the network map of institutions of English literature (Figure 3A), the network temporal node was 65, the network temporal connectivity was 89, and the network density was 0.0428. In the network map of authors of Chinese literature (Figure 3B), the network temporal node was 40, the network temporal connectivity was 4, and the network density was 0.0051.

**Table 3:** The top 10 institutions publishing articles.

No.	English literature		Chinese literature	
	institution	Frequency	institution	Frequency
1	Harvard University	115	Shanghai University of Traditional Chinese Medicine	17
2	Harvard University Medical Affiliates	101	School of Acupuncture and Massage, Chengdu University of Traditional Chinese Medicine	15
3	Beijing University of Chinese Medicine	100	Beijing University of Traditional Chinese Medicine Dongzhimen Hospital	10
4	Kyung Hee University	86	Yueyang Hospital of Integrated Traditional Chinese and Western Medicine Shanghai University of Traditional Chinese Medicine	10
5	Harvard Medical School	85	Zhejiang University of Traditional Chinese Medicine Third Clinical Medical College	10
6	Chengdu University of Traditional Chinese Medicine	83	Institute of Acupuncture, China Academy of Chinese Medical Sciences	9
7	China Academy of Chinese Medical Sciences	82	Shanxi University of Traditional Chinese Medicine	8
8	Shanghai University of Traditional Chinese Medicine	81	Guangzhou University of Traditional Chinese Medicine	7
9	University of California System	70	School of Acupuncture and Massage, Beijing University of Traditional Chinese Medicine	7
10	US Department of Veterans Affairs	62	College of Acupuncture and Moxibustion, Fujian University of Traditional Chinese Medicine	6

### 3.4 Visual mapping of keyword networks

#### 3.4.1 Visual mapping of keyword co-occurrence

**Figure 4:**
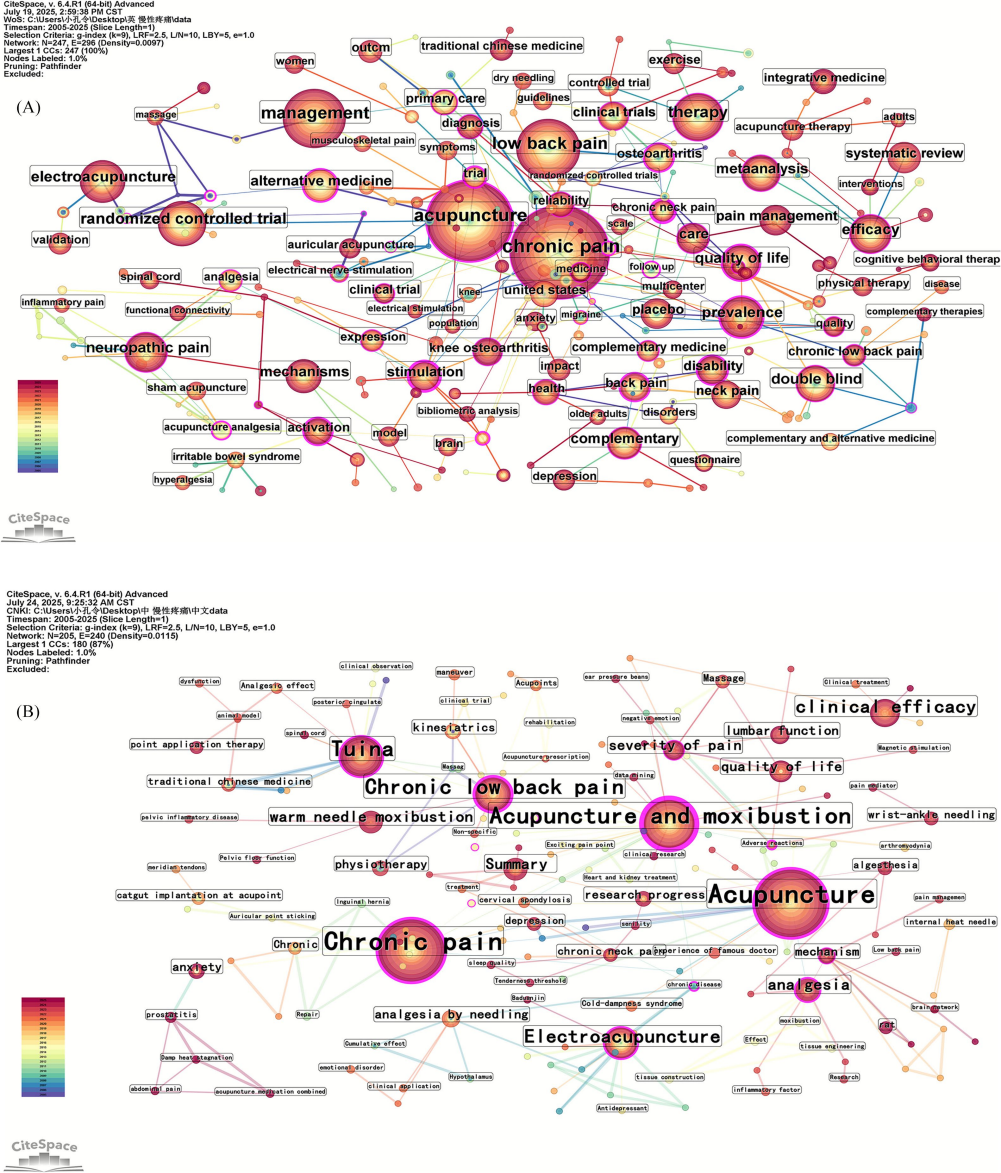
**(A)** Keyword co-occurrence mapping in English literature; **(B)** keyword co-occurrence mapping in Chinese literature.

A higher frequency indicates a higher intensity of research in this field, and a higher centrality demonstrates a higher importance of research in this field. CiteSpace was run to generate keyword co-occurrence maps (Figures 4A,B) and rank the frequency of keywords (Table 4). In the network map of the English literature (Figure 4A), the network temporal node was 247, the network temporal connectivity was 296, and the network density was 0.0097. In the network map of authors of Chinese literature (Figure 4B), the network temporal node was 205, the network temporal connectivity was 240, and the network density was 0.0115.

**Table 4:** The top 10 frequency-ranking keywords.

No.	English literature		Chinese literature	
	Keyword	Frequency	Keyword	Frequency
1	acupuncture	655	Acupuncture	143
2	chronic pain	610	Chronic pain	132
3	low back pain	403	Acupuncture and moxibustion	80
4	management	372	Tuina	60
5	pain	262	Chronic low back pain	53
6	therapy	246	Electroacupuncture	35
7	randomized controlled trial	233	Clinical efficacy	31
8	electroacupuncture	205	Summary	22
9	prevalence	190	Analgesia	20
10	efficacy	184	Warm needle moxibustion	20

#### 3.4.2 Visual mapping of keyword clustering

**Figure 5:**
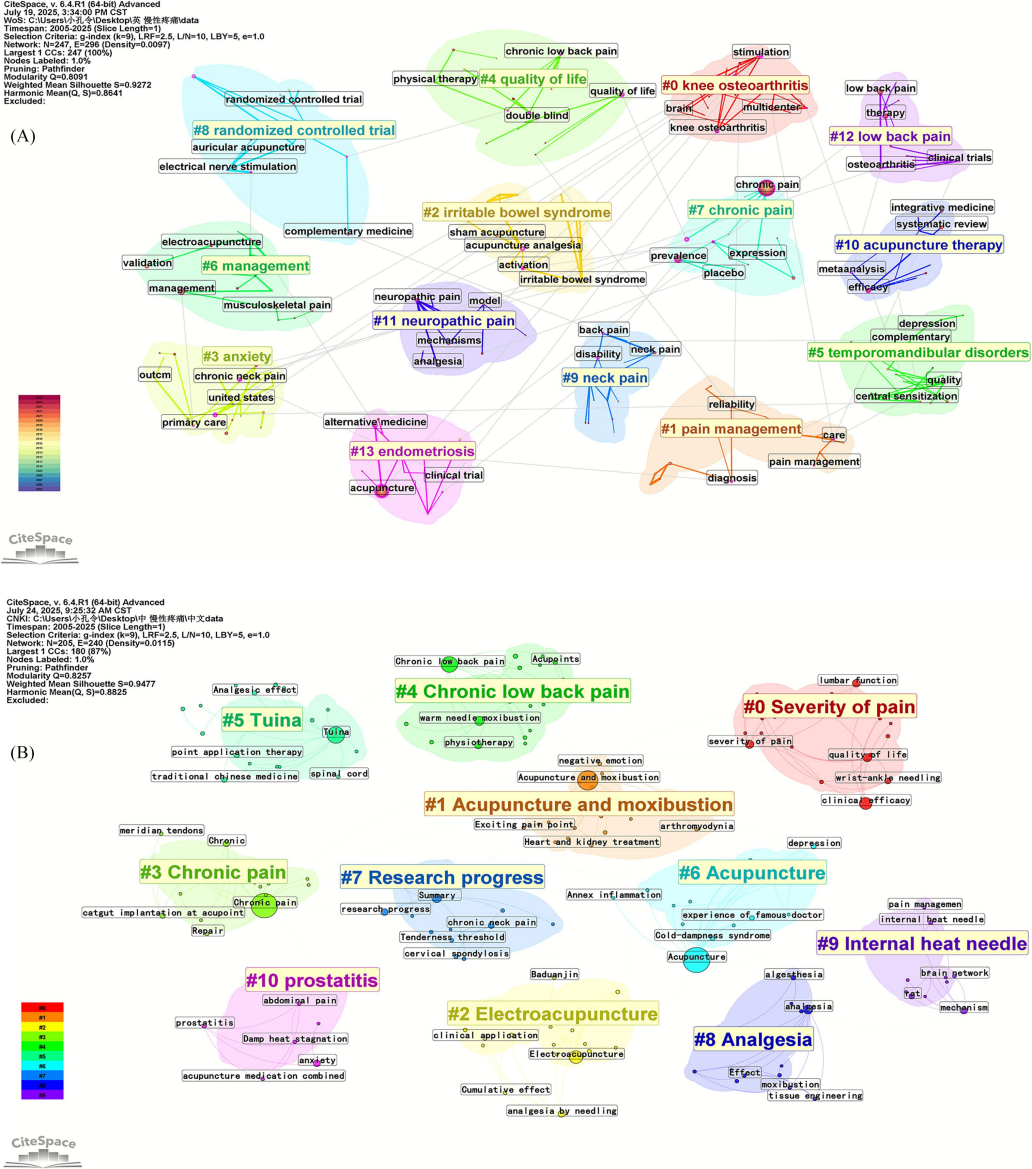
**(A)** Keyword clustering mapping in English literature; **(B)** keyword clustering mapping in Chinese literature.

CiteSpace is used to cluster keywords by extracting keywords from the literature and naming the clusters. The Q value is the modularity value of the clustering, which reflects the modularity of the network; the more significant the value, the better the clustering effect of the network. Q > 0.3 implies that the clustering structure is substantial. The Silhouette clustering average contour value (S) reflects the homogeneity of the network. The closer the value is to 1, the higher the homogeneity of the network. S > 0.5 means the clustering is reasonable, and S > 0.7 is convincing. In the English literature, the keyword clustering map (Figure 5A) had the following values: Q = 0.8091 and S = 0.9272. In the Chinese literature, the keyword clustering map (Figure 5B) had the following values: Q = 0.8257 and S = 0.9477. The results show that the clustering structure of this map is significant and that the clustering results are more credible.

**Figure 6:**
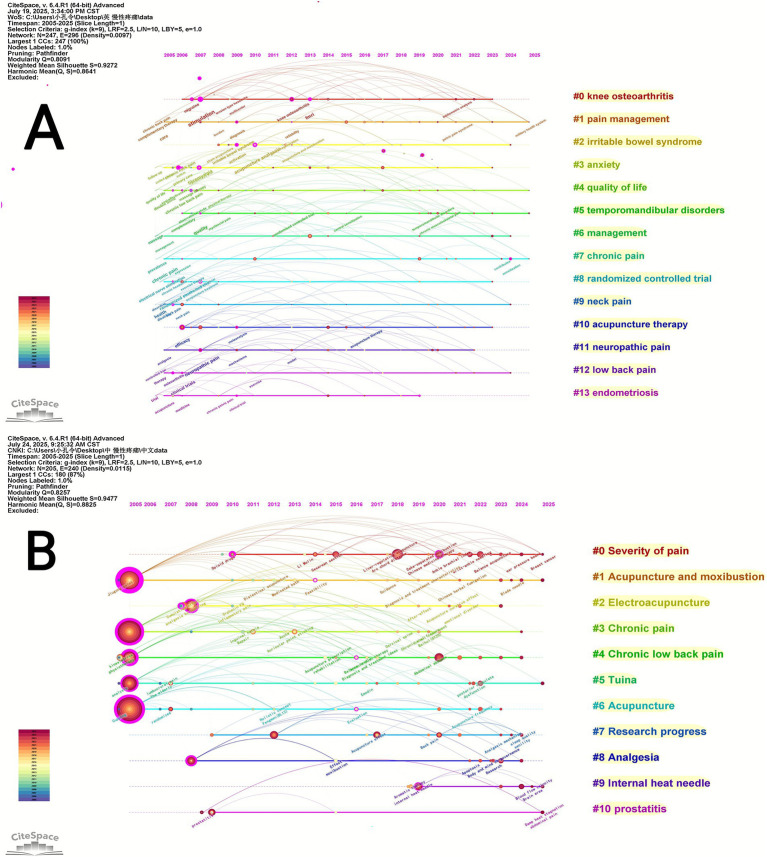
**(A)** Keyword clustering timeline mapping in English literature; **(B)** keyword clustering timeline mapping in Chinese literature.

Keyword timeline mapping can be used to analyze the changes produced by the development of each cluster over time and the connections between clusters. The horizontal axis represents the timeline and the vertical axis represents the cluster ID. Keywords from the Chinese and English literature were clustered for timeline analysis using CiteSpace (Figures 6A,B).

#### 3.4.3 Visual mapping of keyword bursting

**Figure 7:**
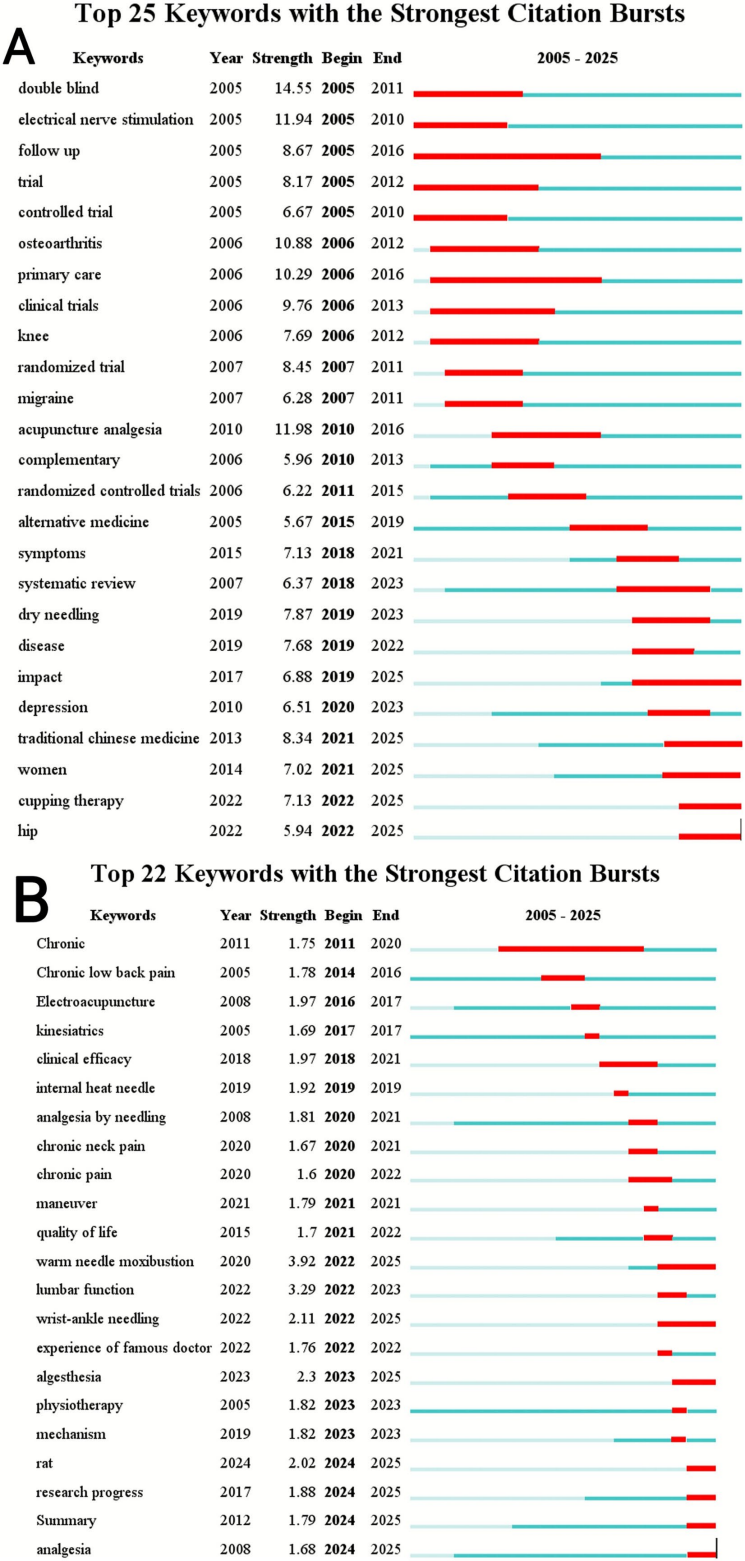
**(A)** Keyword bursting mapping in English literature; **(B)** keyword bursting mapping in Chinese literature.

A bursty keyword is a word frequently mentioned and discussed during a certain period, which indicates that the research area affected by the keyword has become hot or highly concerned at that time. The keywords of the Chinese and English literature were analyzed using the CiteSpace software to generate a visual mapping of keyword bursting (Figures 7A,B).

## 4 Discussion

### 4.1 Research overview

#### 4.1.1 Analysis of annual publication volume

An analysis of relevant English-language literature in this field reveals that published papers increased steadily from 2005 to 2011. The number of publications increased rapidly from 2012 to 2025; in 2020, it reached its peak. The primary focus of discussion was acupuncture therapy and lower back pain. An analysis of relevant Chinese literature in this field shows that the number of Chinese publications increased steadily from 2005 to 2018. From 2019 to 2025, the number of published papers increased rapidly; in 2022, the number of published documents peaked, with discussions primarily focused on acupuncture therapy, tuina therapy, and improvements in lumbar spine function. The overall number of published papers on traditional Chinese external therapies for chronic pain is rising, indicating that this field is increasingly attracting widespread attention from society.

#### 4.1.2 Analysis of author publication volume

An analysis of the publication output of authors in English-language literature reveals that the top three authors by publication volume are Witt, Claudia M, Macpherson, Hugh, and Kong, Jian. Moura, Caroline de Castro, and Fang Jianqiao have collaborated most closely with other authors. An analysis of the publication volume of Chinese literature authors revealed that the top three authors in terms of publication volume were Fang Jianqiao, Kong Lingjun, and Fang Min. Fang Jianqiao, Ma Chao, and Wu Shaoling have the closest collaborative relationships with other authors.

According to Price’s law, the formula for determining core authors is M ≈ 0.749√Nmax, where M equals at least the number of issued articles published by core authors and Nmax denotes the number of published articles of the authors with the highest number of issued articles in the statistical period.

Among the authors of the English literature, Witt, Claudia M had the highest number of publications, that is, Nmax = 47. From the formula, M was ≈5.135 publications; thus, authors with six or more publications in this field are core authors, and there were 304 core authors. The publication volume of core authors accounted for 15.54% of all authors’ publication volume, which is <50%; Among the authors of the Chinese literature, Fang jianqiao has the highest number of publications, that is, Nmax = 10. From the formula, M was ≈2.37; thus, the authors with three or more publications in this field are the core authors, and there were 75 core authors. The publication volume of core authors accounted for 9.61% of all authors’ publication volume, which is <50%. This study summarizes and analyses the publication output of authors in Chinese and English literature within this field and concludes that no stable core group of authors has yet emerged. Collaboration among relevant authors must be strengthened to enhance research quality and foster further development in this domain.

#### 4.1.3 Analysis of institutional publication volume

An analysis of the number of publications by English-language literature institutions reveals that the top three institutions in terms of publication volume are Harvard University, Harvard University Medical Affiliates, and Beijing University of Chinese Medicine. Among these, Harvard Medical School and the University of California System have collaborated extensively with other institutions.

An analysis of the publication volume of Chinese literature institutions reveals that the top three institutions are Shanghai University of Traditional Chinese Medicine, School of Acupuncture and Massage, Chengdu University of Traditional Chinese Medicine, and Beijing University of Traditional Chinese Medicine, Dongzhimen Hospital.

### 4.2 Analysis of keyword networks

Keywords can reveal an article’s main direction. This study summarizes the research hotspots in this field and predicts research trends by analysing the keywords of relevant literature.

According to the analysis by CiteSpace software, the high-frequency keywords in English literature are ‘Acupuncture,’ ‘Chronic pain,’ ‘Low back pain,’ ‘Management,’ ‘Pain,’ and ‘Therapy.’ The high-frequency keywords in Chinese literature are ‘Acupuncture,’ ‘Chronic pain,’ ‘Acupuncture and moxibustion,’ ‘Tuina,’ and ‘Chronic low back pain.’

An analysis of the keyword clustering visualization map of Chinese and English literature revealed that in English literature, #0 knee osteoarthritis, #1 pain management, #2 irritable bowel syndrome, #5 temporomandibular disorders, #9 neck pain, #11 neuropathic pain, #12 low back pain, and #13 endometriosis are common types of chronic pain-related diseases. #3 anxiety is a common psychological state associated with chronic diseases. #4 quality of life and #6 management focus on the impact of the disease on quality of life. #10 acupuncture therapy is a standard treatment method in traditional Chinese medicine external therapy, with electroacupuncture being the most common. In Chinese literature, #1 Acupuncture and moxibustion, #2 Electroacupuncture, #5 Tuina, and #6 Acupuncture are standard treatment methods in traditional Chinese medicine external therapy. #4 Chronic low back and #10 prostatitis are common types of chronic diseases. #0 Severity of pain and #8 Analgesia are related to the efficacy of traditional Chinese external therapies. #7 Research progress primarily summarizes the latest research findings.

An analysis of keyword salience visualization maps for Chinese and English literature was conducted. Over the past two decades, in English literature, early studies primarily focused on ‘double blind,’ ‘electrical nerve stimulation,’ ‘follow-up,’ ‘trial,’ ‘controlled trial,’ ‘osteoarthritis,’ ‘primary care,’ ‘clinical trials,’ and ‘knee’; Mid-term research primarily focused on ‘randomised trial,’ ‘migraine,’ ‘acupuncture analgesia,’ ‘complementary,’ ‘randomised controlled trials,’ ‘alternative medicine,’ and ‘symptoms’; Late-stage research primarily focused on ‘systematic review,’ ‘dry needling,’ ‘disease, “impact, “depression,’ ‘traditional Chinese medicine,’ ‘women,’ ‘cupping therapy,’ and ‘hip’. In Chinese literature, early studies primarily focused on ‘Chronic’, ‘Chronic low back pain,’ ‘Electroacupuncture,’ and ‘Kinesiology’; Mid-term studies mainly focused on ‘Clinical efficacy,’ ‘Internal heat needle,’ ‘Analgesia by needling,’ ‘Chronic neck pain,’ ‘Chronic pain,’ ‘Manoeuvre,’ and ‘Quality of life’; Later studies primarily focused on ‘warm needle moxibustion,’ ‘lumbar function,’ ‘wrist-ankle needling,’ ‘experience of famous doctors,’ ‘Algesthesia,’ ‘Physiotherapy,’ ‘Mechanism,’ ‘Rat,’ ‘research progress,’ ‘Summary,’ and ‘Analgesia.’ Through an analysis of keyword emergence, the research trends in the use of traditional Chinese medicine external therapies for chronic pain are primarily concentrated on cupping therapy, warm needle acupuncture therapy, wrist and ankle acupuncture, and chronic pain accompanied by depressive states.

### 4.3 Hot topics

The research hotspots are mainly focused on electroacupuncture therapy and tuina therapy in traditional Chinese medicine external treatment methods, as well as common chronic pain-related diseases.

#### 4.3.1 Electroacupuncture therapy

In 2020, NICE included acupuncture in its guidelines for the treatment of chronic pain (18, 19). Additionally, relevant clinical trials have confirmed the efficacy of acupuncture for pain relief (8). Electroacupuncture is a form of acupuncture that uses an electroacupuncture device to deliver pulsed electrical currents through fine needles inserted into specific points on the body to prevent and treat diseases. In the clinical treatment of chronic pain, electroacupuncture demonstrates good analgesic effects (20–22). Related clinical studies have confirmed that the long-term clinical efficacy of electroacupuncture is stable, and patients receiving electroacupuncture therapy can gradually reduce their use of opioid medications (23, 24). Regarding the mechanism of action of electroacupuncture therapy for pain relief, relevant animal studies have confirmed that its analgesic effects may be related to the inhibition of iron death in spinal cord neurons, the suppression of microglia activation, the inhibition of the NOD-like receptor protein 3 inflammasome in microglia, and the activation of CB2 receptors in fibroblasts, thereby reducing the expression of IL-1β (25–28). At the same time, researchers conducted a systematic review and meta-analysis of relevant literature, once again confirming the efficacy and safety of electroacupuncture for treating chronic pain (29) clinical practice, patients with chronic pain often experience anxiety and depression. Electroacupuncture therapy, in addition to its analgesic effects, can regulate patients’ psychological states (30). The mechanism may involve regulating the Anterior Cingulate Cortex, modulating the cAMP-response element-binding protein pathway in the spinal cord, restoring the phosphorylation of NR1 in the hippocampus, and activating the rACC-thalamic glutamatergic circuit, etc. (31–33). A literature reviewD1 orsts that the mechanism underlying the antidepressant effects of electroacupuncture may involve electroacupuncture activation of dopamine receptor D1 or inhibition of dopamine receptor D2 in the basolateral amygdala (34). In the clinical application of electroacupuncture for chronic pain, this therapy is often combined with other treatments, such as exercise therapy and traditional Chinese medicine therapy (35–37). In a clinical trial of electroacupuncture for chronic nonspecific low back pain, the clinical efficacy of alternating frequencies was superior to that of high frequencies, regardless of whether the stimulation sites were traditional acupoints or myofascial points (38). Therefore, selecting electroacupuncture frequency and intensity is critical in clinical applications.

#### 4.3.2 Tuina therapy

A social survey revealed that the probability of chronic pain patients seeking massage therapy is 32% (39). Related clinical trials have confirmed that tuina therapy demonstrates good short-term and long-term efficacy in treating chronic pain (40–43). Additionally, after physicians focus on massaging the relevant tissues in the painful areas, patients’ range of limb movement can be significantly improved (44, 45). In treatment protocols for chronic low back pain, this therapy is often combined with exercise therapy and home cupping therapy (46–49). In terms of clinical efficacy, tuina therapy not only provides analgesic effects but also addresses accompanying symptoms in patients with chronic pain, such as constipation, anxiety, and depression (50, 51). Researchers used Proton Magnetic Resonance Spectroscopy to observe changes in brain metabolic products in patients with chronic pain. The results confirmed that spinal manipulation can affect the central nervous system of patients with low back pain and alter brain metabolic products (52). Currently, relevant clinical trials are investigating the most appropriate treatment frequency and duration of single massage therapy sessions to optimize its application in treating chronic pain (53, 54).

#### 4.3.3 Common chronic pain-related diseases

According to the analysis of the CiteSpace visual map, chronic musculoskeletal pain, chronic visceral pain, and chronic neuropathic pain are the types of chronic pain with higher prevalence in clinical practice. Among chronic musculoskeletal pain, the most common types are low back pain, knee osteoarthritis, and neck and shoulder pain; among chronic visceral pain, the most common types are irritable bowel syndrome, endometriosis, and prostatitis.

Epidemiological surveys indicate that the prevalence of chronic musculoskeletal pain increased from 10 to 13.5–47% between 2003 and 2011. Additionally, the prevalence of chronic pain increases with age. Epidemiological surveys show that the prevalence rate among 12-year-olds is 12.5%, rising to 24.1% among 15-year-olds, reaching a peak of 53–54% among those aged 50–74 (55, 56). In addition to age, gender is also a factor influencing the prevalence of chronic pain. Relevant surveys indicate that the prevalence rate among women is higher than that among men; however, the reasons for this phenomenon remain unclear and require further investigation (57). Additionally, joint hypermobility, ulcerative colitis, and respiratory system diseases increase the likelihood of developing chronic musculoskeletal pain disorders (58–61). Chronic musculoskeletal pain most commonly affects the lumbosacral and lumbar spinal segments, followed by the lower and middle cervical spine. Consequently, clinical treatment plans should prioritize therapeutic interventions and preventive measures for these regions (62). Acupuncture demonstrates favourable analgesic effects in treating knee osteoarthritis and chronic low back pain, with particularly pronounced short-term efficacy observed when local tender points.

In 2006, patients with chronic neuropathic pain constituted one-sixth of all chronic pain sufferers, with a prevalence rate of 8.2%. By 2009, this prevalence had risen to 17.9% (63, 64). The primary site of pathology in chronic neuropathic pain patients is predominantly the lower limbs. This condition exhibits higher pain intensity and frequency compared to other chronic pain disorders, and patients are more prone to emotional issues such as depression and anxiety (65, 66). Regarding gender, this condition is more prevalent among women. Relevant social surveys confirm that it is associated with psychological state, labor intensity, and social status (67). Relevant animal studies have confirmed that cupping therapy alleviates chronic neuropathic pain by enhancing the NMDA receptor NR1 (68). Wei, Xiao-Ya et al. demonstrated through clinical trials that acupuncture achieves analgesic effects by modulating neural activity in the right superior parietal lobule in the treatment of chronic neuropathic pain (69).

The aetiology of chronic visceral pain is often attributed to persistent inflammation, abnormal vasoconstriction, and mechanical injury (70). Adolescent stress responses may lead to dysfunction in the central nucleus of the amygdala. This physiological reaction causes abnormal expression of glucocorticoid receptors and corticotropin-releasing hormone. Concurrently, prolonged exposure to high-stress environments enhances pain perception. Both factors consequently elevate the risk of developing chronic pain (71–73). Brain imaging studies in patients with irritable bowel syndrome, functional dyspepsia, and bladder pain syndrome—all chronic visceral pain conditions—reveal abnormal brain responses. These include heightened resting-state brain activity, altered brain connectivity, and matter properties of grey and white matter. Concurrently, abnormal neuronal activity may also contribute to chronic pain (74, 75). Among chronic visceral pain disorders, irritable bowel syndrome and inflammatory bowel disease are relatively common, with a prevalence rate of 15% (76). Research indicates that alterations in visceral fat are associated with chronic visceral pain disorders; consequently, monitoring visceral fat accumulation may effectively prevent such conditions (77). Relevant animal studies have confirmed that moxibustion therapy, a topical treatment method in traditional Chinese medicine, exhibits favourable analgesic effects for chronic visceral pain. Its therapeutic mechanism may be associated with the RNA-miRNA-mRNA network within the spinal cord ring and the mitogen-activated protein kinase signaling pathway in the spinal cord (78–80).

### 4.4 New trends

The research trends have primarily focused on external therapeutic methods in traditional Chinese medicine, including cupping therapy, warm needle acupuncture therapy, wrist-ankle acupuncture therapy, and chronic pain accompanied by depressive states.

#### 4.4.1 Cupping therapy

Cupping therapy is frequently employed in clinical practice to treat chronic neck and lumbar pain. Relevant clinical trials confirm that cupping therapy can increase skin temperature and effectively alleviate patients’ neck pain (81, 82). Patients report a noticeable reduction in neck pain symptoms immediately after a single treatment session (83). For patients enduring pain, altered posture also causes significant disruption to daily life. Lauche, Romy et al. (84) confirmed that cupping therapy alleviates pain and corrects patients’ posture. Regarding long-term efficacy, Lauche, Romy et al. conducted a follow-up survey 2 years after the clinical trial, involving patients who had completed the treatment course. The results indicated that cupping therapy demonstrates favourable long-term efficacy in improving patients’ quality of life and physiological function. Concurrently, relevant clinical trials confirm cupping therapy as a viable home treatment option (85) patients with chronic low back pain, cupping therapy effectively alleviates lumbar pain symptoms, with analgesic effects from a single treatment persisting for up to one week (86, 87). Clinically, this therapy is frequently combined with other modalities (88–90). Regarding clinical efficacy assessment, clinical trials incorporate objective clinical efficacy criteria beyond pain scales, including multi-channel surface electromyography and shear wave elastography (91, 92). Relevant systematic reviews and meta-analyses further confirm the safety and efficacy of cupping therapy for chronic pain (92, 93).

#### 4.4.2 Warm needle acupuncture therapy

Warm needle acupuncture therapy is a therapeutic technique combining needle insertion with moxibustion. In clinical application, this method leverages the advantages of both acupuncture and moxibustion, enhancing its efficacy in dredging the meridian and promoting the circulation of qi and blood. The procedure involves the practitioner inserting fine needles into the treatment area. Once the sensation of qi is achieved, a ball of moxa wool or a suitable moxa cone is placed atop the needle handle. The needles are retained for 15 to 20 min, until the moxa wool or cone completely burns away. Qifei Zhang et al. conducted a clinical randomised trial to evaluate the clinical efficacy of acupuncture-related therapies for chronic pain. The results indicated that warm needle acupuncture demonstrated favourable clinical efficacy in treating chronic pain (94). Concurrently, researchers conducted a meta-analysis of relevant literature in this field, further confirming the safety and efficacy of warm acupuncture in treating chronic pain (95–97).

#### 4.4.3 Wrist-ankle acupuncture therapy

Wrist-ankle acupuncture is a form of superficial subcutaneous needling therapy that treats ailments by inserting needles into corresponding points on the wrists of the upper limbs and the ankles of the lower limbs. Wrist-ankle acupuncture can provide effective pain relief, whether employed alone or in combination with other therapies (98). Concurrently, a clinical study has confirmed that wrist-ankle acupuncture is crucial in exercise programmes for treating chronic neck pain (99). Animal studies suggest that its therapeutic mechanism may involve inhibiting 5-hydroxytryptomaine type 3A receptor expression, thereby alleviating pain perception in rats by enhancing descending pain modulation systems (100). Not only for patients with chronic pain, but also for healthy adults, wrist-ankle acupuncture can elevate the body’s pain threshold 30 to 70 min after treatment (101).

#### 4.4.4 Chronic pain accompanied by depressive states

The severity of chronic pain conditions and the number of affected areas influence patients’ psychological state and daily activities (102–105). Relevant clinical studies confirm that patients with chronic pain frequently experience symptoms of depression and anxiety, with 42 per cent of such patients suffering from depression (106). The severity of the patient’s depression is positively correlated with the intensity of their physical pain (107–109). Research into relevant mechanisms indicates that the anterior cingulate cortex constitutes a pivotal cortical region for human pain perception. In individuals with chronic pain, abnormal expression within the anterior cingulate cortex leads to enhanced long-term potentiation and upregulation of cGMP-dependent protein kinase I at both mRNA and protein levels. This mechanism explains why chronic pain sufferers frequently experience accompanying depressive psychological states (110, 111). Relevant research has confirmed that the dosage of opioid medications correlates with depressive states in patients with chronic pain. Therefore, when formulating clinical management plans for chronic pain, emphasis should be placed on assessing patients’ depressive states (103, 112, 113). Concurrently, both relevant clinical trials and literature analyses have confirmed that acupuncture therapy within the external treatment methods of traditional Chinese medicine can effectively alleviate patients’ depressive symptoms, holding significant clinical practical significance (114–117).

## 5 Strengths and limitations

The use of CiteSpace for literature visualization and analysis was a significant strength in this study. Discussing the findings helps uncover current research hotspots and trends in this field to guide research objectives and provide a reference for researchers, thus providing more reference information and a basis for future research. However, there is a limitation of this study; that is, this study counts the relevant studies up to the current time, but in the future, there will be newly published articles that will not be recorded in this study. Therefore, to address this issue, researchers must update and recapitulate their current status. Nonetheless, the bibliometric methodology used in this study effectively summarizes the field’s current state and provides new informative insights into research hotspots and trends.

## 6 Conclusion

This study comprehensively and objectively analysed relevant literature on applying traditional Chinese medicine external therapies for chronic diseases over the past two decades, generating visualized maps of annual publication volumes, authors, and keywords. Current research indicates promising prospects for development in this field, though academic collaboration remains essential. The research hotspots in this field focus on electroacupuncture and tuina therapy as external treatment methods in Traditional Chinese Medicine and the classification of common chronic pain conditions. Among the categories of chronic pain with higher clinical prevalence are chronic musculoskeletal pain, chronic visceral pain, and neuropathic pain. Consequently, clinical efforts should focus on strengthening the prevention of these conditions and conducting investigations to summarize relevant influencing factors. The research trends have primarily centred on external therapeutic methods in traditional Chinese medicine, including cupping therapy, warm needle acupuncture therapy, and wrist-ankle acupuncture, alongside chronic pain accompanied by depressive states. Therefore, in clinical practice, attention should be paid to enhancing clinical efficacy through the combined application of the aforementioned therapies. Concurrently, this study indicates that relevant researchers should employ traditional Chinese medical theory in formulating clinical protocols to leverage the distinctive advantages of external therapeutic methods. Building upon analgesic efficacy, these approaches should alleviate patients’ depressive states.

## Data Availability

The original contributions presented in the study are included in the article/supplementary material, further inquiries can be directed to the corresponding authors.
